# Age‐related decline in BubR1 impairs adult hippocampal neurogenesis

**DOI:** 10.1111/acel.12594

**Published:** 2017-04-06

**Authors:** Zhongxi Yang, Heechul Jun, Chan‐II Choi, Ki Hyun Yoo, Chang Hoon Cho, Syed Mohammed Qasim Hussaini, Ambrosia J. Simmons, Seonhee Kim, Jan M. van Deursen, Darren J. Baker, Mi‐Hyeon Jang

**Affiliations:** ^1^ Department of Neurologic SurgeryMayo Clinic College of MedicineRochesterMNUSA; ^2^ Department of NeurosurgeryFirst Hospital of Jilin UniversityChangchunChina; ^3^ Department of Anatomy and Cell BiologyShriners Hospital for Children Pediatric Research CenterTemple University Lewis Katz School of MedicinePhiladelphiaPAUSA; ^4^ Department of Biochemistry and Molecular BiologyMayo Clinic College of MedicineRochesterMNUSA; ^5^ Department of Pediatric and Adolescent MedicineMayo Clinic College of MedicineRochesterMNUSA; ^6^ Present address: Medical Scientist Training ProgramIrvine School of MedicineUniversity of CaliforniaIrvineCAUSA; ^7^ Present address: Duke‐NUS Medical School8 College RoadSingapore CitySingapore

**Keywords:** Adult neurogenesis, aging, bubR1, dendrite morphogenesis, hippocampus, progeroid mouse model

## Abstract

Aging causes significant declines in adult hippocampal neurogenesis and leads to cognitive disability. Emerging evidence demonstrates that decline in the mitotic checkpoint kinase BubR1 level occurs with natural aging and induces progeroid features in both mice and children with mosaic variegated aneuploidy syndrome. Whether BubR1 contributes to age‐related deficits in hippocampal neurogenesis is yet to be determined. Here we report that *BubR1* expression is significantly reduced with natural aging in the mouse brain. Using established progeroid mice expressing low amounts of BubR1, we demonstrate these mice exhibit deficits in neural progenitor proliferation and maturation, leading to reduction in new neuron production. Collectively, our identification of BubR1 as a new and critical factor controlling sequential steps across neurogenesis raises the possibility that BubR1 may be a key mediator regulating aging‐related hippocampal pathology. Targeting BubR1 may represent a novel therapeutic strategy for age‐related cognitive deficits.

The hippocampus is one neurogenic niche where new neurons arising from neural stem cells (NSCs) are constantly generated throughout life in a process called adult hippocampal neurogenesis (Ming & Song, 2011). Deficits in this process are observed with aging and are believed to underlie age‐related cognitive deficits (van Praag *et al*., 2005). However, the molecular identity governing such deficits is not fully understood.

A mitotic checkpoint kinase, BubR1, has emerged as a key factor in age‐related pathology and lifespan (Baker *et al*., 2004). Whether BubR1 also regulates age‐related changes in hippocampal neurogenesis is unknown. Notably, *BubR1* is expressed in the postnatal mouse dentate gyrus and is relatively higher in the subgranular zone (SGZ) than the dentate granule layer (Fig. S1A, Supporting information). In addition, *BubR1* is expressed in radial glia‐like NSCs (RGCs; Fig. S1B, Supporting information) and is reduced in an age‐dependent manner (Fig. 1A). We hypothesized that age‐dependent regulation of BubR1 plays a possible role in hippocampal neurogenesis.

**Figure 1:**
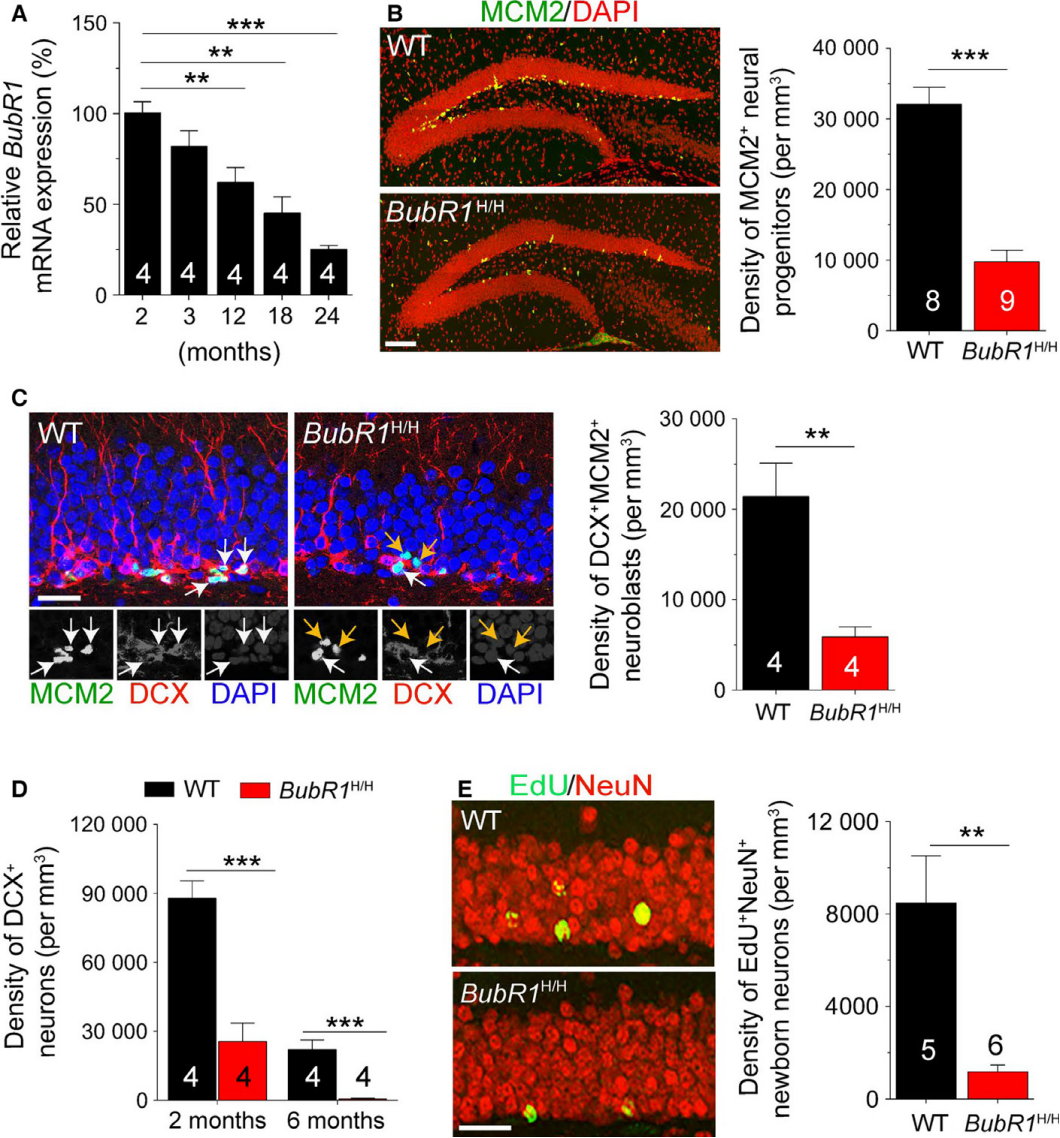
Impaired neural progenitor proliferation and neurogenesis in adult *BubR1* ^H/H^ mice. (A) *BubR1* expression as determined by qRT–PCR. Values are normalized to 2‐month‐old mice. One‐way ANOVA with Bonferroni post hoc test. (B) Sample images of MCM2 and DAPI staining and quantification. Scale bars: 100 μm. (C) Sample images of MCM2, DCX, and DAPI staining and quantification. Scale bar: 50 μm. White arrows point to MCM2^+^ DCX ^+^ neuroblasts, and yellow arrows point to MCM2^+^ DCX ^−^ neural progenitors. (D) *BubR1* ^H/H^ mice show age‐dependent reduction in DCX ^+^ immature neurons. (E) Sample images of EdU and NeuN staining and quantification. Scale bar: 50 μm. All values represent mean ± SEM. **: *P *<* *0.01, ***: *P *<* *0.001, unpaired *t*‐test unless noted otherwise. Number associated with bar graphs indicates the number of mice examined.

Using adult *BubR1* ^H/H^ mice with reduced hippocampal *BubR1* levels (Fig. S1C, Supporting information), we first showed significantly reduced cell proliferation in the SGZ (Fig. 1B) and subventricular zone (Fig. S2B, Supporting information). Progenitor cell types vulnerable to BubR1 insufficiency included significant reductions in activated RGCs (Fig. S2E, Supporting information), intermediate progenitor cells (IPCs; Fig. S2F, Supporting information), and neuroblasts (Fig. 1C). While slightly reduced, quiescent RGCs were not statistically different (Fig. S2D, Supporting information). Furthermore, this decrease was exacerbated in *BubR1* ^H/H^ mice in an age‐dependent manner (Fig. 1D). Notably, cell proliferation was not affected during embryonic day 14 and postnatal day 7 (Fig. S3, Supporting information), indicating deficits seen in *BubR1* ^H/H^ mice were not due to early developmental dysfunction. Subsequently, *BubR1* ^H/H^ mice exhibited a significant decrease in the density of EdU^+^NeuN^+^ mature new neurons (Fig. 1E), while survival of new cells was not affected (Fig. S4, Supporting information). Thus, these results indicate that the reduction in hippocampal neurogenesis may result primarily from a decrease in neural progenitor proliferation, rather than affecting survival.

To further investigate a postmitotic role of BubR1, we examined the localization of BubR1 in postmitotic neurons derived from isolated NSCs *in vitro*. BubR1 localized in the cytoplasm and dendrites (Fig. 2A), suggesting a possible role in maturation or dendrite development of new neurons. At 4 weeks after EdU injection, we found a significant increase in the percentage of DCX^+^NeuN^−^ immature neurons with a concurrent decrease of DCX^−^NeuN^+^ mature neurons in *BubR1* ^H/H^ mice (Fig. 2B), indicating impaired neuronal maturation. To examine impact on dendrite morphogenesis, we utilized a coexpressing GFP and shRNA‐*BubR1* retroviral approach to selectively knock down BubR1 within new neurons (Fig. S5A,B, Supporting information). GFP^+^ new neurons at 14 days postinjection exhibited decreased primary dendrite length, total dendrite length, total branch number, and branch point number with shRNA‐*BubR1* expression compared to shRNA‐control (Fig. 2C). Furthermore, these morphological alterations in BubR1 knockdown were significantly rescued in BubR1‐overexpression mice (excluding total branch number), indicating dendrite morphogenesis is mediated through BubR1 in a cell‐autonomous manner (Fig. S5C, Supporting information).

**Figure 2:**
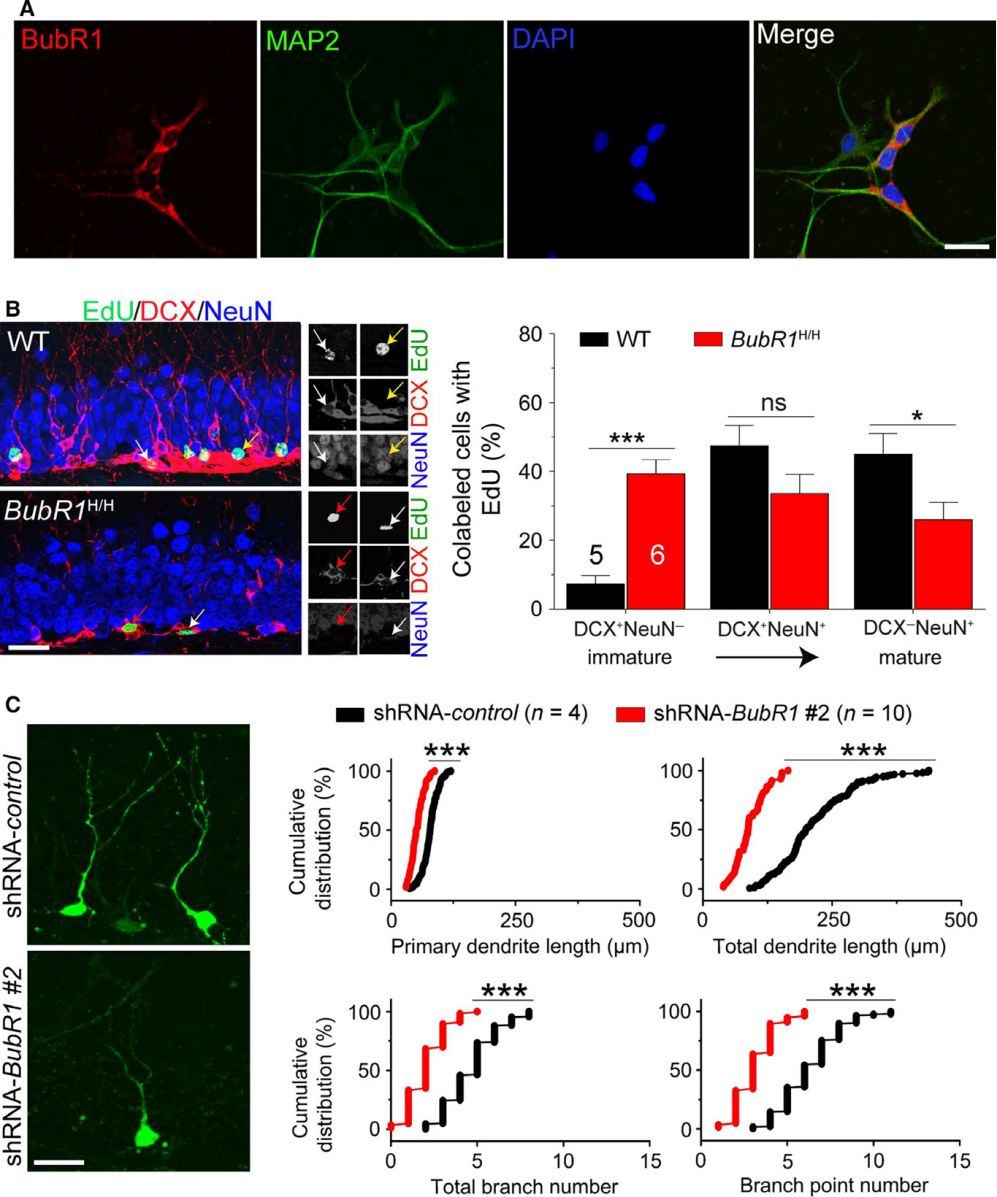
Deficits in neuronal maturation in adult *BubR1* ^H/H^ mice. (A) BubR1 expression in postmitotic neurons *in vitro*. Sample images of MAP2 (a mature neuron marker), BubR1, and DAPI staining in differentiated postmitotic neurons derived from hippocampal NSCs. Scale bar: 20 μm. (B) Impaired neuronal maturation in the adult *BubR1* ^H/H^ mice. Sample images of EdU^+^ DCX ^+^NeuN^−^ immature new neurons (red arrows), EdU^+^ DCX ^+^NeuN^+^ intermediate new neurons (white arrows), and EdU^+^ DCX ^−^NeuN^+^ mature new neurons (yellow arrows). Scale bar: 50 μm. All values represent mean ± SEM. *: *P *<* *0.05, **: *P *<* *0.01, ns: no significance, unpaired *t*‐test. (C) Impaired dendrite morphogenesis of new neurons by BubR1 knockdown. Left: Sample confocal images of GFP ^+^ neurons. Scale bar: 20 μm. Right: Cumulative distribution plots of dendrite analysis of new neurons. Each symbol represents data from a single GFP ^+^ neuron. ***: *P *<* *0.001, Kolmogorov–Smirnov test.

In this study, we have identified several novel functions of BubR1 in the adult brain. First, we show *BubR1* level is significantly reduced with age. Given that BubR1 insufficiency contributes to age‐related pathology including short lifespan (Baker *et al*., 2004), our findings extend the established function of BubR1 to aging and cognitive decline. Second, BubR1 is primarily known as a key regulator for mitosis (Kapanidou *et al*., 2015). We identify an adult‐specific mitotic function of BubR1 in ensuring a precise number of neural progenitors are proliferated and an effective rate of neurogenesis is maintained. Third, we show a critical postmitotic function of BubR1. Rather than affecting cell survival, BubR1 insufficiency impairs neuronal maturation and impairs dendrite morphogenesis. Interestingly, a previous study observed BubR1 knockdown resulting in increased dendrite growth examined at postnatal day 2 (Watanabe *et al*., 2014), while we found the opposite phenotype in 8‐week‐old mice. This discrepancy may be due to different local microenvironment properties between the early and later postnatal dentate gyrus, resulting in differential regulation of dendritic growth (Kim *et al*., 2012). Considering defects in neuronal maturation are associated with cognitive dysfunction (van Praag *et al*., 2005), it is conceivable that age‐related BubR1 decline may contribute to cognitive aging. Furthermore, BubR1 is reported to be a spindle assembly checkpoint kinase involved in cell cycle progression and arrest, and with dysfunction, may lead to cell aneuploidy (Kapanidou *et al*., 2015). It is possible impaired new neuron development may result from aneuploidy in new neurons although it remains an open question for future study.

## Author contributions

Z.Y., H.J., and M‐H.J. designed research; Z.Y., H.J., C‐I.C., S.M.Q.H., K.H.Y., and C.H.C. performed research; A.S. and S.K. provided embryonic data; J.M.v.D. and D.J.B. provided BubR1 expression data; Z. Y., H.J., S.M.Q.H., and M‐H.J. wrote the manuscript.

## Funding

Mayo Clinic Center for Regenerative Medicine, Whitehall Foundation.

## Conflict of interest

The authors declare no competing interests.

## Supporting information


**Fig. S1** BubR1 expression in adult dentate gyrus.
**Fig. S2** Impaired neural progenitor proliferation in adult *BubR1* ^H/H^ mice.
**Fig. S3** Neural progenitor proliferation during early development in *BubR1* ^H/H^ mice.
**Fig. S4** No difference for cell survival was found in *BubR1* ^H/H^ mice.
**Fig. S5** BubR1 knockdown impairs dendrite morphogenesis of new neurons.
**Table S1** Antibody list.
**Data S1** Materials and methods.
