# Supplementary material for: Age‐related decline in BubR1 impairs adult hippocampal neurogenesis
Source: Aging Cell. 2017 Apr 6;16(3):598–601. doi: 10.1111/acel.12594 (PMC5418205; doi:10.1111/acel.12594)
Supplement: Supplementary file 1 — Fig. S1 BubR1 expression in adult dentate gyrus. Fig. S2 Impaired neural progenitor proliferation in adult BubR1 H/H mice. Fig. S3 Neural progenitor proliferation during early development in BubR1 H/H mice. Fig. S4 No difference for cell survival was found in BubR1 H/H mice. Fig. S5 BubR1 knockdown impairs dendrite morphogenesis of new neurons. Table S1 Antibody list. Data S1 Materials and methods. [file ACEL-16-598-s001.docx]

**Supporting Information**

**Age-related decline in BubR1 impairs adult hippocampal neurogenesis**

Zhongxi Yang, Heechul Jun, Chan-II Choi, Ki Hyun Yoo, Chang Hoon Cho, Syed Mohammed Qasim Hussaini, Ambrosia Simmons, Seonhee Kim, Jan M. van Deursen, Darren J. Baker, Mi-Hyeon Jang

**Supporting Information includes**

Detailed materials and methods

**Fig. S1.** BubR1 expression in adult dentate gyrus

**Fig. S2.** Impaired neural progenitor proliferation in adult *BubR1*^H/H^ mice

**Fig. S3.** Neural progenitor proliferation during early development in *BubR1*^H/H^ mice

**Fig. S4.** No difference for cell survival was found in *BubR1*^H/H^ mice

**Fig. S5.** BubR1 knockdown impairs dendrite morphogenesis of new neurons

**Table S1.** Antibody list

**Materials and Methods**

**Mice**

BubR1 hypomorphic (*BubR1*^H/H^) mice were generated as described previously ([Baker *et al.* 2004](#_ENREF_2)). *BubR1*^H/H^ mice were backcrossed to the C57BL/6 background for over 10 generations. While complete knockout of *BubR1* gene causes early embryonic lethality ([Wang *et al.* 2004](#_ENREF_17)), *BubR1*^H/H^ mice exhibited normal brain size at birth, but underwent delayed postnatal development ([Baker *et al.* 2004](#_ENREF_2)), resulting in a smaller brain size (Fig. S1D). Mice were housed in standard cages under a 12 h light/dark cycle with lights on at 7:00 AM. Food and water were provided *ad libitum*. Animal care and handling procedures were approved by the Mayo Clinic Institutional Animal Care and Use Committee (IACUC) in accordance with National Institutes of Health guidelines.

***In situ* hybridization**

*In situ* hybridization was performed using the QuantiGene (QC) ViewRNA kit from Panomics as previously described with slight modifications ([Cajigas *et al.* 2012](#_ENREF_3)). Briefly, hippocampal tissue was embedded in paraffin block and sectioned at a thickness of 5 µm. mRNA sequence was designed by Affymetrix (Santa Clara, CA). Sections were fixed with 4% PFA at room temperature for 30 min. After completion of *in situ* hybridization, sections were incubated in blocking buffer (4% goat serum in PBS) for 1 hour, and then immunostained with GFAP antibody. DAPI staining was used as counterstaining. The sections were imaged using a Zeiss LSM780 confocal microscope (Figs. S1A and S1B).

**Quantitative RT-PCR (qRT-PCR)**

Total RNA was isolated from cerebellum (Fig. 1A) or hippocampal tissue (Fig. S1C) samples using the RNAeasy mini kit (Qiagen). For cDNA synthesis, SuperScript III Reverse Transcriptase (Life Technologies) was used according to the manufacturer’s protocol. qRT-PCR was performed using the SYBR-green Real-Time Mastermix (BioRad). Initial 5 minute denaturing was followed by 40 cycles of denaturing at 95°C for 15 sec, and annealing/elongation at 60°C for 1 min. Either β-actin or GAPDH was used as an internal control. The following BubR1 primer sequences were used: 5'-CCAGCTGAAGGTTGAGGGAG-3' (forward), 5’-TGAAGTGTGGACATGACCCG-3’ (reverse).

**EdU labeling, immunohistochemistry, confocal imaging, and analysis**

Adult female *BubR1*^H/H^ and their WT littermates at 7-10 weeks of age were used. After post-fixation and cryoprotection, coronal brain sections (40 µm in thickness) were prepared in serial order for a total of ~50 sections using a microtome (Leica SM 2010R), which is along the anterior-posterior axis. Approximately 4 sections in each brain sample from WT and *BubR1*^H/H^ mice were selected in serial order of dentate gyrus from the anterior to posterior axis, and processed for immunostaining as previously described ([Jang *et al.* 2013](#_ENREF_8)). For immunostaining with anti-MCM2, anti-nestin, anti-Tbr2 and anti-DCX presented in Figs. 1B-1D, Figs. S2B-S2F, an antigen retrieval protocol was performed using a microwave as previously described ([Hussaini *et al.* 2013](#_ENREF_7); [Jang *et al.* 2013](#_ENREF_8)). Briefly, citrate buffer (1.8 mM citric acid, 8.2 mM tri-sodium citrate) was pre-heated for 5 minutes at maximum power. Sections were then placed in hot citrate buffer and incubated for another 7 minutes at maximum power. Sections were then allowed to cool at room-temperature in citrate buffer for 1 hour. Sections were immunostained with each set of combination of markers. Antibodies used in this study are listed in Table S1. All z-stack images in entire dentate gyrus were acquired on a Zeiss LSM 780 single-photon confocal system using a multi-track configuration to confirm double labeled cells. Double labeled cells were quantified using Zen software (Zeiss). Quiescent radial glial cells (qRGCs) were defined by nestin^+^MCM2^-^ with radial processes (Fig. S2D), activated RGCs defined by nestin^+^MCM2^+^ with radial processes (Fig. S2E), intermediate progenitor cells (IPCs) were defined by Tbr2^+^MCM2^+^ cells (Fig. S2F), and neuroblasts were defined by MCM2^+^DCX^+^ cells (Fig. 1C) as previously stated ([Hussaini *et al.* 2013](#_tyjcwt); [Jang *et al.* 2013](#_3dy6vkm)). Stereological quantification of marker+ cells in the SGZ and granule cell layer were carried out as we used previously with slight modification ([Kempermann *et al.* 1997](#_ENREF_9); [Jang *et al.* 2013](#_ENREF_8)). Briefly, each type of neural progenitor along the SGZ was counted to obtain an absolute cell number. In each of the counted dentate gyri, the dentate gyrus area was measured from the middle z-plane, by manually tracing along its edge and enclosing it using the area measurement feature provided by the Zen software (Zeiss). The volume of the dentate gyrus section was calculated by multiplying the area by its thickness. The cell count was divided by the resultant section volume to obtain the total cell density in the dentate gyrus per mm^3^.

To quantify neurogenesis and neuronal maturation presented in Fig. 1E, and Fig. 2B, mice were injected with EdU (41.1 mg/kg body weight, i.p.) once daily for 5 days and sacrificed 4 weeks after the first EdU injection. Approximately 4 sections in each brain sample from WT and *BubR1*^H/H^ mice were selected in serial order of dentate gyrus from the anterior to posterior axis, and processed for immunostaining with anti-DCX and anti-NeuN. All z-stack images in the entire dentate gyrus were acquired to confirm double labeled cells. Immature neurons were defined by EdU^+^DCX^+^NeuN^-^ cells, intermediate neurons from immature neuron to mature neuron were defined by EdU^+^DCX^+^NeuN^+^ cells, and mature neurons were defined by EdU^+^DCX^-^NeuN^+^ cells. To analyze neuronal maturation, EdU^+^DCX^+^NeuN^-^, EdU^+^DCX^+^NeuN^+^, and EdU^+^DCX^-^NeuN^+^ cells were counted between WT and *BubR1*^H/H^ mice using a Zen software (Zeiss). Percentage of each stage of neurons out of total new neuron number (immature + intermediate + mature neurons) was calculated ([Jang *et al.* 2013](#_ENREF_8)).

**Construction and lentiviral production**

A retroviral vector pUEG co-expressing shRNA under the U6 promoter and GFP under the EF1α promoter were used (Fig. S5A) ([Duan *et al.* 2007](#_ENREF_4)). The short-hairpin sequences used were as follows: (shRNA-*BubR1* #1) 5’-AAGGGAAGCCGAGCTGTTGAC-3’; (shRNA-*BubR1* #2) 5’- AGATCCTGGCTAACTGTTC-3’. To validate the knockdown efficiency of the shRNA at the protein level, shRNA-*BubR1* (#1 and #2) or shRNA-*control* were transfected into HEK293T cells, which are known to highly express BubR1 *in vitro* ([Miyamoto *et al.* 2011](#_ENREF_14)), followed by Western blot analysis using anti-BubR1 antibody (Fig. S5B). Actin was used as a loading control. Values were normalized to shRNA-*control* sample.

**Engineered retrovirus production, stereotaxic surgery, and dendrite analysis**

Engineered self-inactivating murine retroviruses were used to co-express GFP and shRNA-*BubR1* specifically in proliferating cells and their progeny as previously described ([Faulkner *et al.* 2008](#_ENREF_5); [Ma *et al.* 2009](#_ENREF_11)). High titers of engineered retroviruses were produced by co-transfection of retroviral vectors and VSVG into HEK293gp cells followed by ultra-centrifugation of viral supernatant. Retroviruses co-expressing shRNA-*BubR1* (#1 and #2) or shRNA-control and GFP were stereotaxically injected into the dentate gyrus of 6-8-week old adult C57BL/6 WT mice at 4 sites (0.5 µl per site at 0.25 µl/min) with the following coordinates (in mm): posterior = -2 from Bregma, lateral = ± 1.6, ventral = 2.5; posterior = -3 from Bregma, lateral = ± 2.6, ventral = 3.2 under anesthesia. Mice were sacrificed at 14 days post viral injection for morphological analysis (Fig. 2C). Complete GFP^+^ neurons with dendritic processes were imaged by confocal microscopy (LSM 780). Z-stack images were projected and semi-automatically traced with NIH ImageJ using the NeuronJ plugin ([Meijering *et al.* 2004](#_ENREF_12); [Schneider *et al.* 2012](#_ENREF_15)). The primary dendrite length, branch point number, total dendrite length and total branch number of each individual GFP^+^ neurons were subsequently analyzed (Fig. 2C) as described in previous report with slight modification ([Gontier *et al.* 2015](#_ENREF_6)). In shRNA-control injected group, 167 neurons were analyzed for primary dendrite length and branch point number, and 178 neurons were analyzed for total dendrite number and branch number. In shRNA-*BubR1* injected group, 57 neurons were analyzed for primary dendrite length and branch point number, and 58 neurons were analyzed for total dendrite number and branch number. For rescue experiment of impaired dendrite morphogenesis seen in shRNA-*BubR1* injected group, we performed stereotaxic injection with shRNA-control and shRNA-*BubR1 #2* retroviruses into the dentate gyrus of 16-week-old BubR1 overexpression (*BubR1*^T23^ line) and WT littermates ([Baker *et al.* 2013](#_ENREF_1)). At 14 days post retroviral injection, injected mice were analyzed for primary dendrite length, total dendrite length, branch point number, and total branch number (Fig. S5C). In shRNA-*control* injected WT group, 42 neurons were analyzed. In shRNA-*BubR1* injected WT group, 31 neurons were analyzed. In shRNA-*control* injected BubR1 overexpression group, 47 neurons were analyzed. In shRNA-*BubR1* injected BubR1 overexpression group, 38 neurons were analyzed. Statistical significance was assessed using the Kolmogorov-Smirnov test (*: *P*<0.05, **: *P*<0.01, ***: *P*<0.001).

**Neuronal differentiation *in vitro***

One-week-old C57BL/6 mice were deeply anesthetized by an overdose of ketamine and decapitated. The brains were removed from the skull and were placed in cold PBS (Life Technologies) and neural precursors derived from dentate gyrus were isolated as described previously ([Song *et al.* 2002](#_ENREF_16)). For neural differentiation, neural precursors were plated onto the Geltrex™ (Life Technologies) coated coverslip with 0.1% FBS-containing proliferation media. The medium was replaced with fresh medium the next day. Subsequently, the cultures were fed with new medium without mitogens (FGF2 and EGF) everyday by replacing 50% of the medium. After 7-10 days of differentiation, cells were fixed in 100% methanol for further staining and analysis (Fig. 2A). Immunostaining was processed with anti-BubR1 (BD), and rabbit polyclonal anti-MAP2 (Table S1).

**Cell survival analysis *in vivo***

To determine whether decreased neuronal production in adult *BubR1*^H/H^ mice is associated with reduced survival of newborn cells, mice were injected with EdU (41.1 mg/kg body weight, i.p.) once a day for 5 days and analyzed 2 and 4 weeks later (Fig. S4A). To determine the survival rate, the total number of EdU^+^ cells at 4 weeks to that of 2 weeks was quantified and the percentage of EdU^+^ cells was compared between WT and *BubR1*^H/H^ mice (Fig. S4B) as described previously ([Lee *et al.* 2011](#_ENREF_10)).

**Statistics**

All statistical analysis was performed using GraphPad Prism 7.00. An unpaired *t*-test (i.e. Student’s *t*-test) was performed to determine statistical significance for two groups. To assess statistical significance for more than two groups, one-way ANOVA was performed with Bonferroni post-hoc test for multiple comparisons. Kolmogorov-Smirnov test was performed for comparisons of cumulative distribution of primary dendrite length, total dendritic length, branch number and branch point number. Outliers on dendrite analysis were identified *via* GraphPad Prism, and these values were removed. Statistical significance was defined as *P*<0.05.

**Supplementary Figures**

**
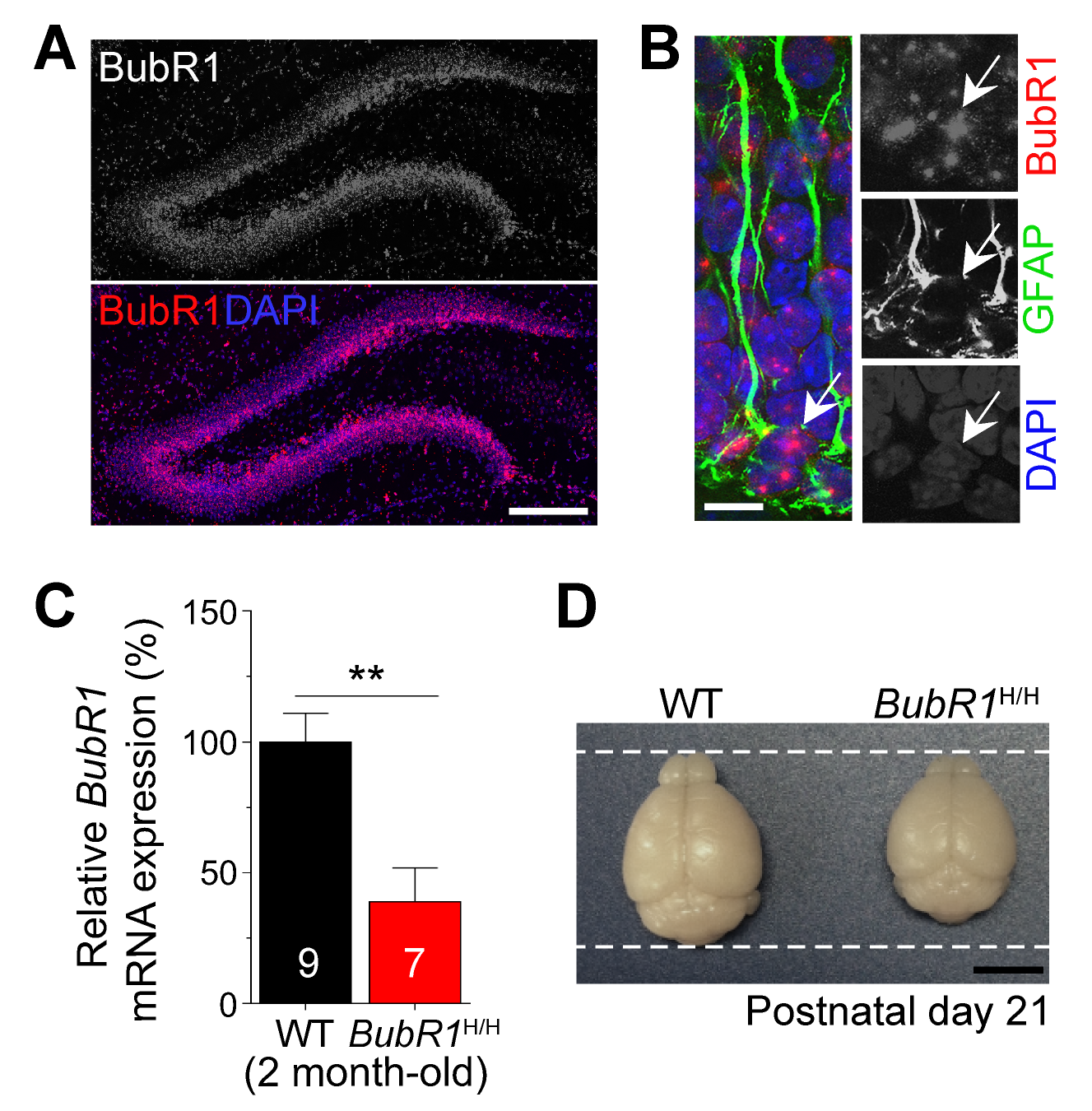
**

**Fig. S1. BubR1 expression in adult dentate gyrus**

**(A)** BubR1 expression in postnatal 21 mouse dentate gyrus by *in situ* hybridization. Scale bar: 200 µm. **(B)** *BubR1* expression in RGCs which is defined by GFAP expressing cells with radial process within the SGZ (white arrow). Scale bar: 10 µm. **(C)** In adult *BubR1*^H/H^ mice, qRT-PCR of hippocampal *BubR1* mRNA reveals *BubR1* expression is significantly reduced. Values are normalized to 2-month-old WT mice. Value represents mean ± SEM. Unpaired *t*-test was used (**: *P*<0.01). The number associated with bar graphs indicates the number of animals examined. **(D)** General characterization of *BubR1*^H/H^ mice. *BubR1*^H/H^ mice show a reduced brain size at postnatal day 21 that persists throughout adulthood. Scale bars: 0.5 cm.


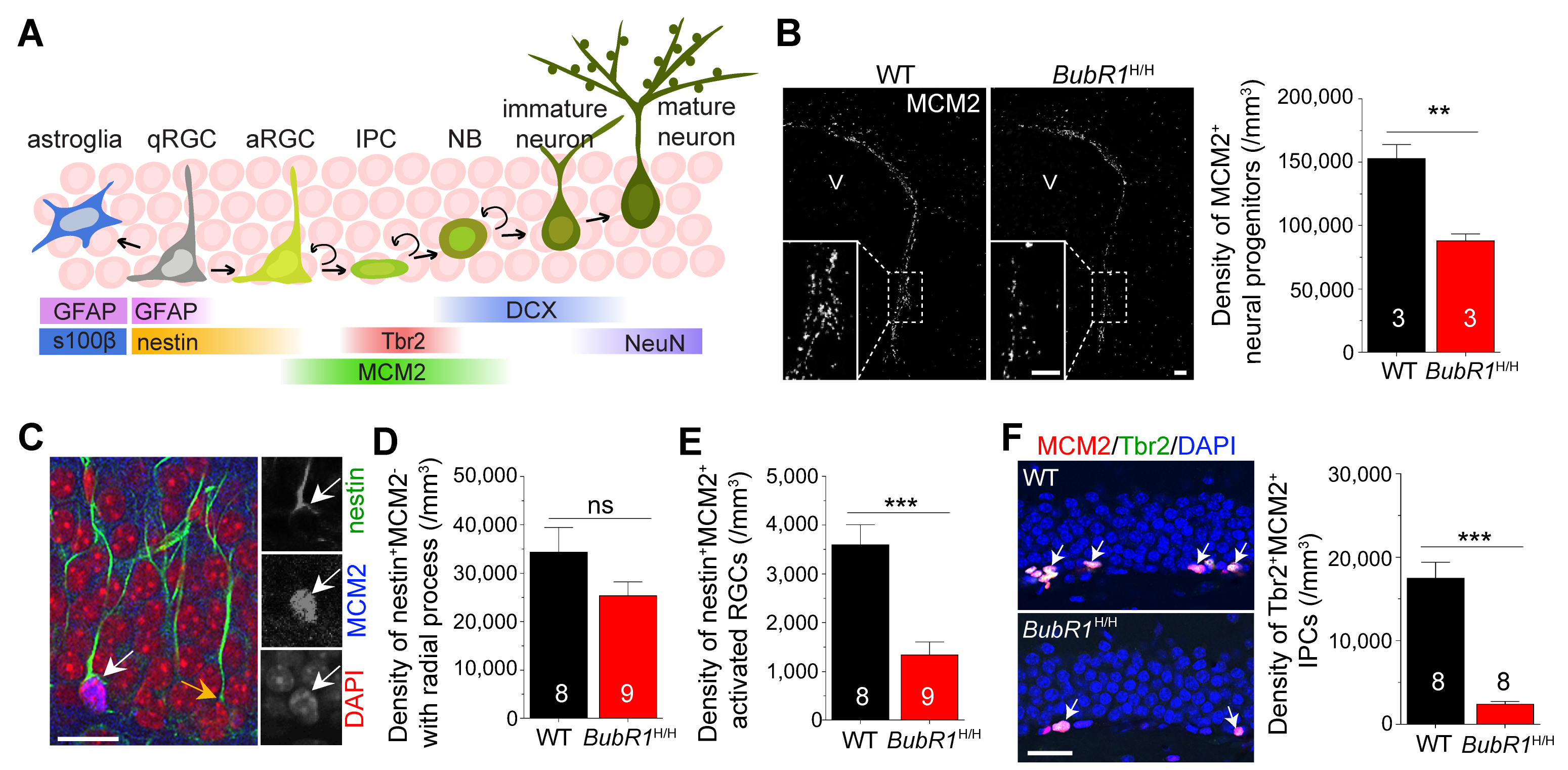


**Fig. S2. Impaired neural progenitor proliferation in adult *BubR1*^H/H^ mice.**

**(A)** Schematic diagram showing cell-type specific markers during adult hippocampal neurogenesis ([Ming & Song 2011](#_ENREF_13)). MCM2: a marker for endogenous cell proliferation, nestin: a marker for neural stem cell, Tbr2: a marker for IPC, DCX: a marker for immature neuron, NeuN: a marker for mature neuron, GFAP with radial process within SGZ: a marker for RGC. GFAP with star-shape morphology: a marker for astrocyte, and s100b: a marker for astrocyte. RGC: radial glial-like neural stem cell, IPC: intermediate progenitor cell, and NB: neuroblast. **(B)** Deficits of neural progenitor proliferation in the subventricular zone (SVZ) of the adult *BubR1*^H/H^ mice. Left: Representative images of MCM2 immunostaining in the SVZ. Inset: a high-magnification view of MCM2^+^ cells. Scale bars: 100 µm each. V; ventricle. Right: Quantification of neural progenitors in the SVZ. Proliferating neural progenitor were significantly reduced in *BubR1*^H/H^ mice. **: *P*<0.01, Unpaired *t*-test. **(C-E)** Quantification of quiescent and activated RGCs. **(C)** Sample images of quiescent RGC (yellow arrow) and activated RGC (white arrow). Scale bar: 50 µm. **(D)** Quiescent RGCs were slightly reduced in *BubR1*^H/H^ mice, but no statistical difference. *P*=0.106, ns; no significance, unpaired *t*-test. **(E)** Quantification of activated RGCs. **(F)** Left: Representative images of MCM2, Tbr2, and DAPI staining in the dentate gyrus. White arrows point to proliferating IPCs defined by MCM2^+^Tbr2^+^ cells. Scale bar: 25 µm. Right: Quantification of IPCs. IPCs were significantly reduced in *BubR1*^H/H^ mice. Note that BubR1 insufficiency mostly susceptible to a population of aRGCs, IPCs and neuroblasts, but not qRGCs. **: *P*<0.01, ***: *P*<0.001, ns: no significance, unpaired *t*-test. All values represent mean ± SEM. The number associated with bar graphs indicates the number of animals examined.


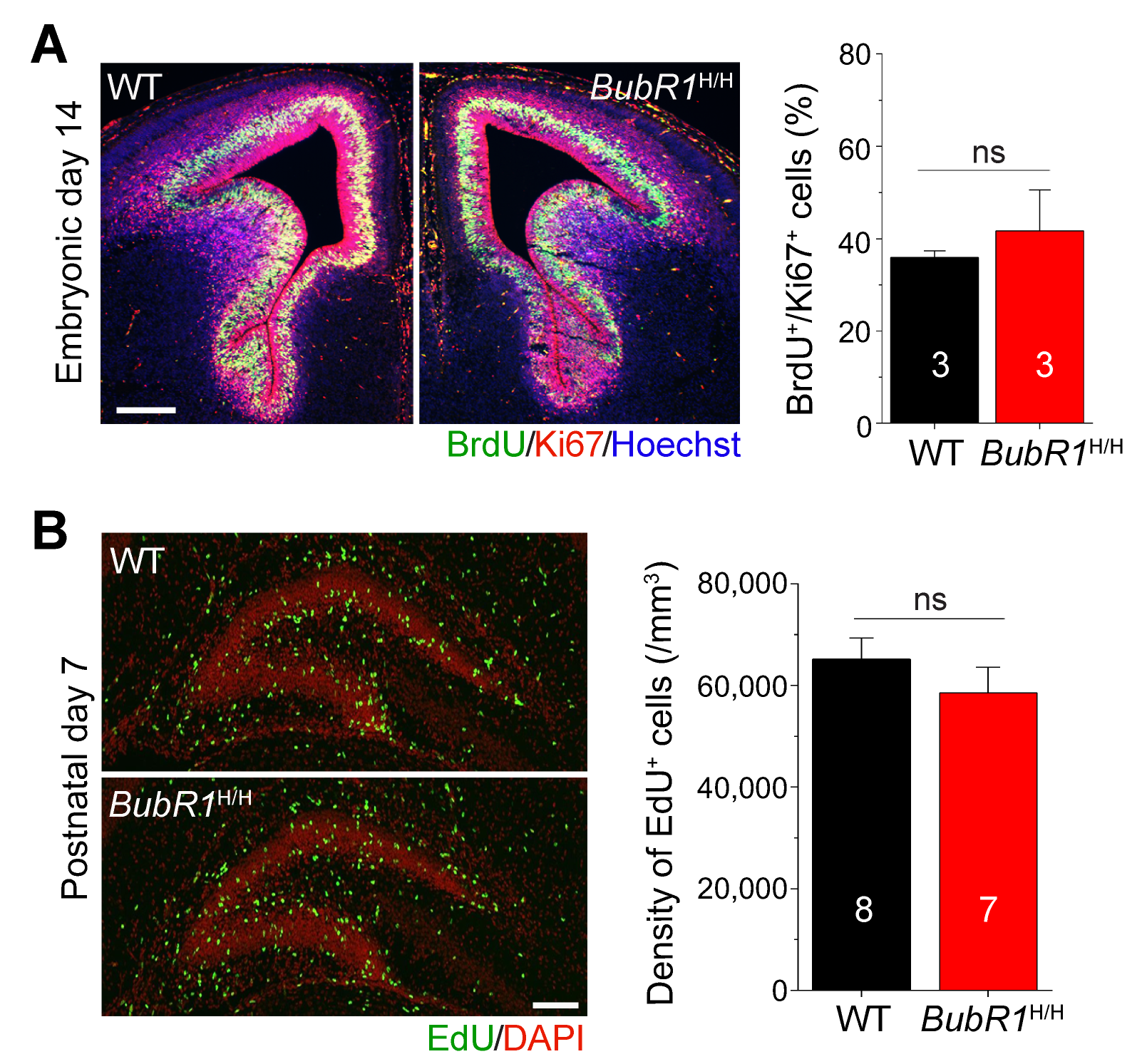


**Fig. S3. Neural progenitor proliferation during early development in *BubR1*^H/H^ mice**

**(A)** *BubR1*^H/H^ mice show no defects in neural progenitor proliferation during embryonic neural development (embryonic day 14). Left: Representative images of BrdU (green), Ki67 (red), and Hoechst (counterstaining; blue). Scale bar: 250 μm. Right: Quantification of the ratio of BrdU to Ki67 cells at E14. **(B)** Normal proliferation continues in *BubR1*^H/H^ mice at postnatal day 7, as measured by EdU with 2 hour pulse chase. Left: Representative images of EdU (green), and DAPI (counterstaining; red). Scale bar: 100 μm. Right: Quantification of the EdU^+^ cells at P7. All values represent mean ± SEM. ns: no significance, unpaired *t*-test.


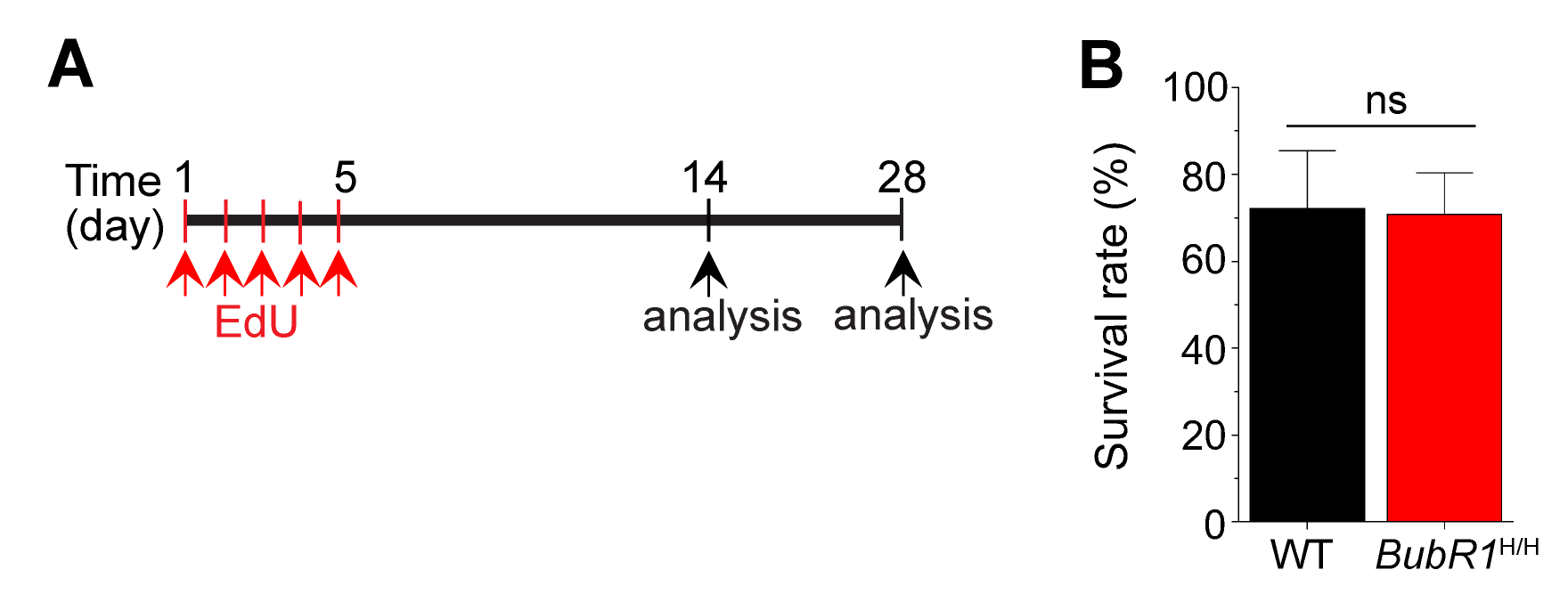


**Fig. S4. No difference for cell survival was found in *BubR1*^H/H^ mice.**

**(A)** Experimental timeline of EdU injections for cell survival analysis. **(B)** The percentage of EdU^+^ cells between 2 and 4 weeks after EdU injection. The percentage of EdU^+^ cells was not significantly different between WT and *BubR1*^H/H^ mice, indicating that BubR1 insufficiency does not impair survival of newly generated neurons during this period of time. Value represents mean ± SEM. ns: no significance, unpaired *t*-test. 8 WT and 6 *BubR1*^H/H^ mice for 2 weeks, and 5 WT and 6 *BubR1*^H/H^ mice for 4 weeks were used.


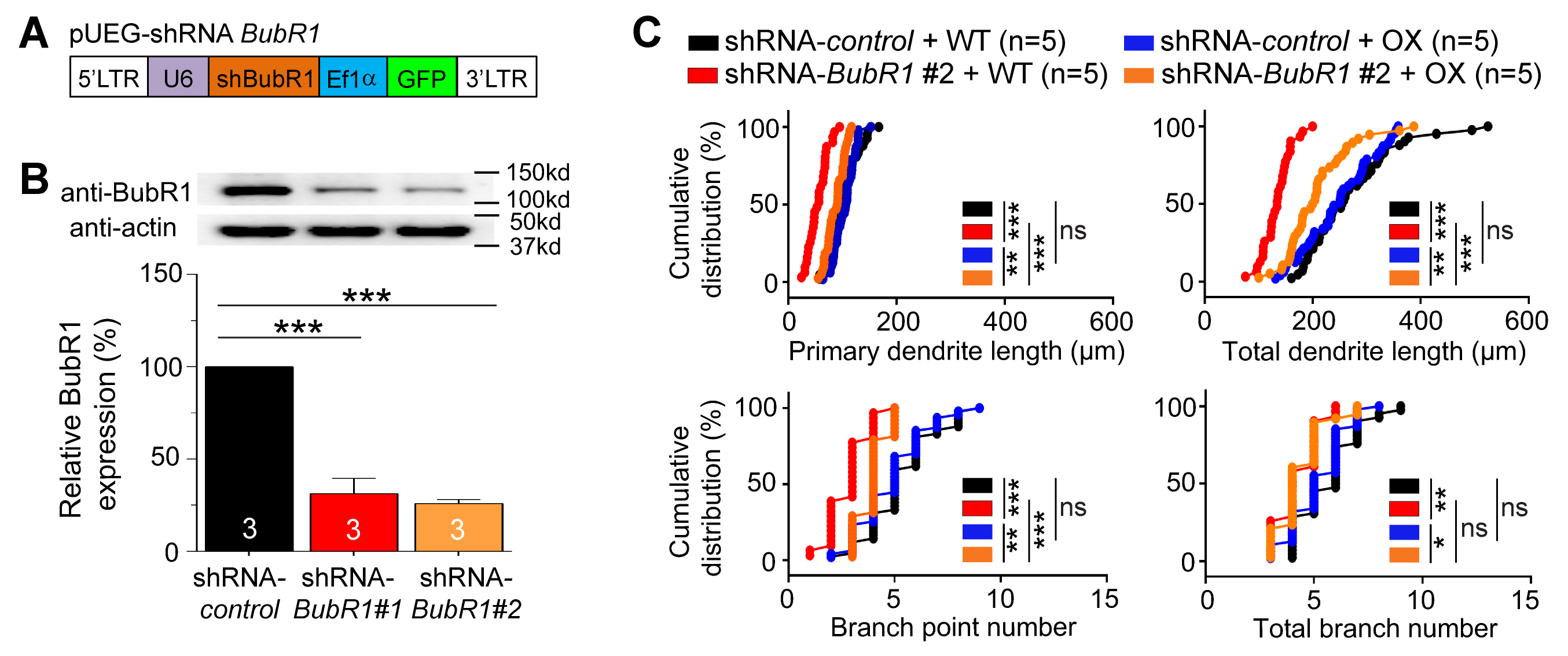


**Fig. S5. BubR1 knockdown impairs dendrite morphogenesis of new neurons.**

**(A-B)** Validation of knockdown efficiency of shRNA-*BubR1* constructs. **(A)** A schematic diagram of the retroviral vectors used (pUEG). **(B)** Constructs expressing different BubR1 shRNAs were transfected into HEK293T cells *in vitro* and equal amount of cell lysate samples were subjected to western blot analysis for BubR1 and β-actin. A sample blot is shown above, and densitometry quantification is shown below. Values represent mean ± SEM. One-way ANOVA was performed with Bonferroni post-hoc test for multiple comparisons (***: *P*<0.001). The number associated with bar graphs indicates the number of animals examined. **(C)** Deficits in dendrite morphogenesis of new neurons by BubR1 knockdown were rescued by BubR1 overexpression (OX). Cumulative distribution plots of primary dendrite length, total dendrite length, branch point number, and total branch number of new neurons under different conditions are shown. Each symbol represents data from a single GFP^+^ neuron. Kolmogorov-Smirnov test was performed. *: *P*<0.05, **: *P*<0.01, ***: *P*<0.001, ns: no significance.

**Table S1. Antibodies used in this study.**

| **Antigen** | **Supplier** | **Catalog Number** | **Host** | **Dilution** |
| --- | --- | --- | --- | --- |
| MCM2 | BD Lab | 610701 | Mouse | 1:500 |
| NeuN | Millipore | MAB377 | Mouse | 1:250 |
| nestin | Aves | NES | Chicken | 1:250 |
| GFAP | Santa Cruz | sc6170 | Rabbit | 1:1000 |
| Tbr2 | Abcam | Ab23345 | Rabbit | 1:500 |
| DCX | Santa Cruz | sc8066 | Goat | 1:500 |
| GFP | Rockland | 600-101-215 | Goat | 1:1000 |
| MAP2 | Chemicon | AB5622 | Rabbit | 1:500 |
| Ki67 | Millipore | AB9260 | Rabbit | 1:200 |
| BrdU | BD Lab | 612503 | Mouse | 1:1000 |
| BubR1 | Abcam | ab6326 | Rat | 1:200 |
| BubR1 | BD Lab | 612503 | Mouse | 1:200 |
| b-actin | Cell Signaling | #12262 | Mouse | 1:2000 |
| DAPI | Invitrogen | D3571 | N.A. | 1:2000 |
| Hoechst | Thermo Fisher | 33342 | N.A. | 1:1000 |
|  |  |  |  |  |
|  |  |  |  |  |
|  |  |  |  |  |

**References**

Baker DJ, Dawlaty MM, Wijshake T, Jeganathan KB, Malureanu L, van Ree JH, Crespo-Diaz R, Reyes S, Seaburg L, Shapiro V, Behfar A, Terzic A, van de Sluis B, van Deursen JM (2013). Increased expression of BubR1 protects against aneuploidy and cancer and extends healthy lifespan. *Nat Cell Biol*. **15**, 96-102.

Baker DJ, Jeganathan KB, Cameron JD, Thompson M, Juneja S, Kopecka A, Kumar R, Jenkins RB, de Groen PC, Roche P, van Deursen JM (2004). BubR1 insufficiency causes early onset of aging-associated phenotypes and infertility in mice. *Nat Genet*. **36**, 744-749.

Cajigas IJ, Tushev G, Will TJ, tom Dieck S, Fuerst N, Schuman EM (2012). The local transcriptome in the synaptic neuropil revealed by deep sequencing and high-resolution imaging. *Neuron*. **74**, 453-466.

Duan X, Chang JH, Ge S, Faulkner RL, Kim JY, Kitabatake Y, Liu XB, Yang CH, Jordan JD, Ma DK, Liu CY, Ganesan S, Cheng HJ, Ming GL, Lu B, Song H (2007). Disrupted-In-Schizophrenia 1 regulates integration of newly generated neurons in the adult brain. *Cell*. **130**, 1146-1158.

Faulkner RL, Jang MH, Liu XB, Duan X, Sailor KA, Kim JY, Ge S, Jones EG, Ming GL, Song H, Cheng HJ (2008). Development of hippocampal mossy fiber synaptic outputs by new neurons in the adult brain. *Proc Natl Acad Sci U S A*. **105**, 14157-14162.

Gontier G, George C, Chaker Z, Holzenberger M, Aid S (2015). Blocking IGF Signaling in Adult Neurons Alleviates Alzheimer's Disease Pathology through Amyloid-beta Clearance. *J Neurosci*. **35**, 11500-11513.

Hussaini SM, Jun H, Cho CH, Kim HJ, Kim WR, Jang MH (2013). Heat-induced antigen retrieval: an effective method to detect and identify progenitor cell types during adult hippocampal neurogenesis. *J Vis Exp*.

Jang MH, Bonaguidi MA, Kitabatake Y, Sun J, Song J, Kang E, Jun H, Zhong C, Su Y, Guo JU, Wang MX, Sailor KA, Kim JY, Gao Y, Christian KM, Ming GL, Song H (2013). Secreted frizzled-related protein 3 regulates activity-dependent adult hippocampal neurogenesis. *Cell Stem Cell*. **12**, 215-223.

Kempermann G, Kuhn HG, Gage FH (1997). More hippocampal neurons in adult mice living in an enriched environment. *Nature*. **386**, 493-495.

Lee MH, Wang T, Jang MH, Steiner J, Haughey N, Ming GL, Song H, Nath A, Venkatesan A (2011). Rescue of adult hippocampal neurogenesis in a mouse model of HIV neurologic disease. *Neurobiol Dis*. **41**, 678-687.

Ma DK, Jang MH, Guo JU, Kitabatake Y, Chang ML, Pow-Anpongkul N, Flavell RA, Lu B, Ming GL, Song H (2009). Neuronal activity-induced Gadd45b promotes epigenetic DNA demethylation and adult neurogenesis. *Science*. **323**, 1074-1077.

Meijering E, Jacob M, Sarria JC, Steiner P, Hirling H, Unser M (2004). Design and validation of a tool for neurite tracing and analysis in fluorescence microscopy images. *Cytometry A*. **58**, 167-176.

Ming GL, Song H (2011). Adult neurogenesis in the mammalian brain: significant answers and significant questions. *Neuron*. **70**, 687-702.

Miyamoto T, Porazinski S, Wang H, Borovina A, Ciruna B, Shimizu A, Kajii T, Kikuchi A, Furutani-Seiki M, Matsuura S (2011). Insufficiency of BUBR1, a mitotic spindle checkpoint regulator, causes impaired ciliogenesis in vertebrates. *Hum Mol Genet*. **20**, 2058-2070.

Schneider CA, Rasband WS, Eliceiri KW (2012). NIH Image to ImageJ: 25 years of image analysis. *Nat Methods*. **9**, 671-675.

Song H, Stevens CF, Gage FH (2002). Astroglia induce neurogenesis from adult neural stem cells. *Nature*. **417**, 39-44.

Wang Q, Liu T, Fang Y, Xie S, Huang X, Mahmood R, Ramaswamy G, Sakamoto KM, Darzynkiewicz Z, Xu M, Dai W (2004). BUBR1 deficiency results in abnormal megakaryopoiesis. *Blood*. **103**, 1278-1285.
